# Usefulness of E7 mRNA in HPV16-Positive Women to Predict the Risk of Progression to HSIL/CIN2+

**DOI:** 10.3390/diagnostics11091634

**Published:** 2021-09-07

**Authors:** Cristina Martí, Lorena Marimón, Ariel Glickman, Carla Henere, Adela Saco, Natalia Rakislova, Aureli Torné, Jaume Ordi, Marta del Pino

**Affiliations:** 1 Institute Clinic of Gynaecology, Obstetrics, and Neonatology, Hospital Clínic, University of Barcelona, 08036 Barcelona, Spain; MARTI@clinic.cat (C.M.); glickman@clinic.cat (A.G.); carlahenere@gmail.com (C.H.); atorne@clinic.cat (A.T.); 2 Institut de Salut Global de Barcelona (ISGlobal), Hospital Clínic, University of Barcelona, 08036 Barcelona, Spain; lorena.marimon@isglobal.org (L.M.); rakislova@clinic.cat (N.R.); jordi@clinic.cat (J.O.); 3 Department of Pathology, Hospital Clínic, University of Barcelona, 08036 Barcelona, Spain; masaco@clinic.cat; 4 Institut D’investigacions Biomèdiques August Pi i Sunyer (IDIBAPS), Hospital Clínic, University of Barcelona, 08036 Barcelona, Spain

**Keywords:** HPV 16, mRNA, E7, HSIL/CIN2+

## Abstract

Objective: To evaluate whether E7 mRNA can predict the risk of progression in women with HPV16 infection. Design: A prospective observational study. Setting: A tertiary university hospital. Population: A cohort of 139 women referred to colposcopy for an abnormal screening result fulfilling the following inclusion criteria: (1) a positive test result confirming HPV16 infection; (2) a biopsy sample with a histological diagnosis of an absence of lesion or low-grade SIL/CIN grade1 (LSIL/CIN1); (3) no previous HPV vaccination; (4) no pregnancy; and (5) no previous cervical treatments; and (6) no immunosuppression. Methods: At the first visit, all women underwent a cervical sample for liquid-based cytology, HPV testing and genotyping, and HPV16 E7 mRNA analysis and a colposcopy with at least one colposcopy-guided biopsy. Follow-up visits were scheduled every six months. In each control, a liquid-based Pap smear, HPV testing, as well as a colposcopy examination with biopsy if necessary were performed. Main outcome measures: Histological diagnosis of HSIL/CIN2+ at any time during follow-up. Results: E7 mRNA expression was positive in 55/127 (43.3%) women included in the study and seven (12.7%) progressed to HSIL/CIN2+. In contrast, only 1/72 (1.4%) women with no HPV16 E7 mRNA expression progressed (*p* = 0.027). HPV16 E7 mRNA expression was associated with a 10-fold increased risk of progression (HR 10.0; 95% CI 1.2–81.4). Conclusions: HPV16 E7 mRNA could be useful for risk stratification of women with HPV16 infection in whom a HSIL/CIN2+ has been ruled out. Funding: Instituto de Salud Carlos III (ICSIII)-Fondo de Investigación Sanitaria and ERDF ‘One Way to Europe’ (PI17/00772).

## 1. Introduction

According to the current guidelines [1,2], women with infection by human papillomavirus 16 (HPV16) warrant immediate colposcopy because of the high risk of harboring high-grade squamous intraepithelial lesion/cervical intraepithelial neoplasia grade 2 or worse (HSIL/CIN2+). However, the management and follow-up of patients with HPV16 infection in whom HSIL/CIN2+ is adequately ruled out is not well defined. Indeed, the risk of this subset of women developing HSIL/CIN2+ is still substantial over time compared with women testing negative [3,4]. However, this risk is variable from woman to woman [1,5,6,7]. Thus, although it is clear that all women who are positive for HPV16 need to be closely followed, only a small percentage actually develop an HSIL/CIN2+ [8]. Stratifying the risk of developing a lesion in these women is particularly challenging. It is a priority to identify biomarkers in cervical cancer screening able to provide information on the risk of progression in these women with persistent HPV16 infection in whom HSIL/CIN2+ has been excluded.

It has been suggested that HPV infections resulting in HSIL/CIN2+ are characterized by specific molecular alterations. However, the molecular profile defining infection at risk of progression has not been well characterized [9,10,11], and currently, it is not possible to identify which women with HPV infection will develop HSIL/CIN2+ before this event has already occurred; therefore, patient-tailored management is not yet a feasible alternative to standard clinical follow-up.

E7 mRNA expression induces abnormal cellular proliferation, transformation, and immortalization, leading to the development of HSIL/CIN2+ [12,13]. Previous studies have shown that the HPV E7 mRNA test has a high clinical sensitivity and specificity to identify women with HPV infection with underlying HSIL/CIN2+ lesions [14,15,16], and the surrogate biomarker of E7 expression p16 is recommended and widely used in the diagnosis of HPV-associated premalignant and malignant lesions [17]. However, to our knowledge, no previous study has determined the prognostic value of HPV E7 mRNA expression in women with HPV16 infection in whom HSIL/CIN2+ has been excluded.

The aim of this study was to evaluate the usefulness of HPV16 E7 mRNA expression to predict women at higher risk of progression from among those with HPV16 infection in whom HSIL/CIN2+ was been carefully ruled out. We have specifically focused on patients with HPV16 infection because of the particularly high risk women with this infection present.

## 2. Material and Methods

### 2.1. Study Population and Case Selection

This was a prospective study conducted at the referral Colposcopy Unit of the Hospital Clinic of Barcelona from October 2011 to September 2015. All women referred to our Colposcopy Unit for an abnormal screening result (a positive high-risk HPV test and/or abnormal Pap-test) within the previous six months were eligible for the study. Women fulfilling the following criteria at the baseline visit were included in the study: (1) a positive test result confirming HPV16 infection; (2) a biopsy sample with a histological diagnosis of absence of lesion or low-grade SIL/CIN grade1 (LSIL/CIN1); (3) no previous HPV vaccination; (4) no pregnancy; (5) no previous cervical treatments for intraepithelial lesions; and (6) no human immunodeficiency virus (HIV) infection or other cause of immunosuppression. Exclusion criteria were: (1) insufficient material for HPV genotyping and E7 mRNA detection; and (2) absence of follow-up.

### 2.2. Cervical Sampling, Colposcopy Evaluation, and Biopsy Collection

A cervical sample was obtained from all women and was preserved in methanol-based fixative (PreservCyt solution, Hologic Corp, Marlborough, MA, USA). This material was used for liquid-based cytology, HPV testing and genotyping, and HPV16 E7 mRNA analysis. After the samples were obtained, all women underwent digital colposcopy and at least one colposcopy-guided biopsy.

Colposcopy was performed using an Olympus EvisExera II CV-180 (Tokyo, Japan) by an experienced colposcopist after application of 5% acetic acid with cotton balls for 1 to 2 min. Colposcopy findings were described following the criteria of the International Federation for Cervical Pathology and Colposcopy (Río de Janeiro 2011) [18,19].

During the colposcopy evaluation, one to four biopsies were obtained from different abnormal areas or from different regions in one large complex abnormal area of the cervix. If no abnormal areas were identified or if less than four colposcopy-directed biopsies were taken, a random biopsy (non-targeted biopsy) from apparently normal epithelium obtained within the transformation zone was also taken [6,20]. Endocervical curettage using a Kervokian curette was performed in all women with a non-completely visible transformation zone. Thus, at least one sample was obtained for histological evaluation in all patients.

### 2.3. Liquid-Based Cytology

The Thin-Prep T2000 slide processor (Hologic) was used to prepare thin-layer cytology slides, which were stained using the Papanicolaou method. A cytotechnologist evaluated the cytology slides using the revised Bethesda nomenclature [21], and then an expert gynecological pathologist revised the result. The samples were subsequently centrifuged, and the pellets were stored at −80 °C before further processing [6].

### 2.4. DNA Isolation, HPV Detection and Genotyping

DNA isolation was performed using the QIAmp MinElute Virus Spin kit (QIAGEN Inc., Valencia, CA, USA) according to the manufacturer’s protocol [22]. Briefly, 10 μL of the isolated DNA were amplified by GP5+/6+ polymerase chain reaction (PCR) and 5 μL of GP5+/6+ amplifiers were used for HPV detection by the enzyme immunoassay (Diassay, Rijswijk, The Netherlands). The cut-off value to classify samples as positive for HPV was three-fold the mean optical density of the PCR-negative controls, which had an optical density ≤0.120 [6].

Individual HPV genotypes of enzyme immunoassay-positive samples were identified using the commercially available LMNX Genotyping kit HPV GP HR (Labo Bio-medical Products B.V. Rijswijk, The Netherlands) according to the manufacturer’s instructions [22,23]. Briefly, the biotinylated PCR products were hybridized to HPV type-specific probes attached to color-coded beads, targeting 18 HPV types: HPV 16, 18, 26, 31, 33, 35, 39, 45, 51, 52, 53, 56, 58, 59, 66, 68, 73, and 82 [6]. Only women with HPV16 infection were included in the study, and the samples were further processed for HPV16 E7 mRNA detection.

### 2.5. RNA Isolation and Quantitative Reverse Transcriptase Polymerase Chain Reaction

Once cytological evaluation and the DNA HPV testing had been performed, samples with HPV16 infection were processed for mRNA isolation and reverse transcriptase polymerase chain reaction (RT-PCR). Five mL of the cell suspension from the cytological samples were centrifuged at 300× *g* for 5 min. RNA extraction was performed using the RNeasy Mini Kit (QIAGEN) with QIAzol-chloroform and subsequent purification over commercial columns following the manufacturer’s protocol [24,25]. The concentration, purity, and integrity of the isolated RNA was determined with Nanodrop ND-1000 (Thermo Scientific, Wilmington, DE, USA). The total RNA concentration ranged from 53 to 753.0 ng/μL, reflecting the variability in cell number in the specimens. A total of 500 ng of total RNA were used in a 20 μL reaction volume for the cDNA synthesis reaction using a high capacity cDNA RT kit (Applied Biosystems, Foster City, CA, USA) according to the manufacturer’s protocol. To exclude DNA contamination, a reaction without RT was run in parallel with each specimen, as described previously [25].

HPV16 E7 mRNA primers and probes were specifically designed to capture all transcripts of the HPV16 E7 gene without risk of cross reactivity with E7 expression of any other HPV types. HPV16 E7 mRNA detection was performed by quantitative PCR (qPCR), using 7500 Real-Time PCR Systems (Applied Biosystems). All qPCRs were performed in triplicate at a reaction volume of 25 μL containing 5 μL of cDNA, diluted at a 1:5 ratio, and mixed with Taqman Universal PCR MasterMix (Applied Biosystems). The following protocol was used for all assays: denaturation (95 °C for 10 min) and amplification (95 °C for 20 s, 60 °C for 1 min) repeated for 40 cycles. The housekeeping genes *GUSB* (beta glucuronidase) and *PGK1* (cGMP-dependent protein kinase 1) were selected as reference genes for quality control of the RNA specimens. This combination of reference genes demonstrated a high stability in expression between groups of normal samples versus HSIL samples [24]. The number of cycles required for the signal to cross the threshold (cycle threshold [Ct] value) for target genes was set at 35 cycles and automatically calculated and recorded by the High-Resolution Melt Software v2.0 for each reaction. Ct levels are inversely proportional to the amount of target nucleic acid in the sample (the lower the Ct level, the greater the amount of target nucleic acid in the sample). For the reference genes *GUSB* and *PGK1*, a Ct value above 35 cycles indicates poor RNA quality. Samples above these Ct values were therefore considered invalid and excluded from the analysis. Among the valid samples, those with a Ct value below 35 cycles for any of the probes used for HPV16 E7 mRNA were considered positive for E7 expression [26,27]. The primer and probe sequences used in the qPCR are listed in Table 1. All reactions were run in singleplex.

### 2.6. Histological Diagnosis

Biopsy samples were fixed in 10% neutral buffered formalin and embedded in paraffin following routine procedures. In all cases, 4 µm sections were stained with hematoxylin and eosin and using the CINtec histology kit for p16 (clone E6H4; mtm-Roche Laboratories, Heidelberg, Germany), following the manufacturer’s protocol. Immunohistochemical staining was performed with the Autostainer Link 48 (Dako Co., Carpinteria, CA, USA), using the EnVision system (Dako). Cases with continuous block staining of cells of the basal and parabasal layers were considered as positive [28].

The final histological diagnosis was based on hematoxylin and eosin and p16 staining. Biopsy specimens were classified as normal, LSIL/CIN1, HSIL/CIN2, HSIL/CIN3, or adenocarcinoma in situ according to the Lower Anogenital Squamous Terminology Standardization Project for HPV-Associated Lesions (LAST) criteria [17]. Positive block staining for p16 in the dysplastic area was required for the diagnosis of HSIL/CIN2+ [17].

### 2.7. Follow-Up Protocol

Follow-up visits were scheduled every six months, as previously described [6]. In each control, a cervical sample was obtained from all women, which was preserved in PreservCyt solution (Hologic). A liquid-based Pap smear, HPV testing, as well as a colposcopy examination were performed in each control.

In case of worsening of the cytological result during follow-up, new colposcopy-directed biopsies, random biopsies of the transformation zone, and/or endocervical curettage were obtained. Colposcopy-directed biopsies were also repeated when worsening of the colposcopy pattern was identified during follow-up.

Women with a confirmed histological diagnosis of HSIL/CIN2-3 or adenocarcinoma in situ (HSIL/CIN2+) underwent excisional treatment by loop excision of the transformation zone and abandoned the study. Patients in whom all the tests became negative during follow-up returned to routine screening.

### 2.8. Categorization of the Patients at the Final Follow-Up Control

Progression was defined as a histological diagnosis of HSIL/CIN2+ at any time during follow-up. Persistence was defined as the presence of HPV16 with a negative LSIL/CIN1 histological result, independently of the Pap test result at the last follow-up control. Regression was defined as a negative result for HPV16, independently of the cytological result and the histological diagnosis (with the exception of HSIL/CIN2+) [6]. Thus, a positive test for HPV other than HPV16 at the end of follow-up was considered as regression.

### 2.9. Statistical Analysis

Data analysis was performed using SPSS version 20.0 software (SPSS, Inc., Chicago, IL, USA). All data related to mRNA expression were analyzed as Ct. Categorical variables are presented as an absolute number and percentages and were compared using the χ^2^ or Fisher exact test. Continuous variables are presented as mean and standard deviation (SD) and were compared using the analysis of variance test. Kaplan–Meier curves and Cox models were used to analyze the risk estimation of progression to HSIL/CIN2+ at the end of follow-up. *p* values less than 0.05 were considered statistically significant.

## 3. Results

A total of 139 women fulfilled the inclusion criteria. Five cases were excluded because of insufficient material or poor RNA quality for E7 mRNA assessment and seven women were lost to follow-up. Thus, 127 women were considered as adequate for analysis and were finally included in the study. The mean age of the overall group was 36.8 ± 11.7 years.

The mean follow-up period was 35.2 ± 21.5 months. Progression to HSIL/CIN2+ during follow-up was confirmed in 8/127 women (6.3%); the mean time from the baseline visit to progression was 22.0 ± 18.8 months. Persistence of HPV16 infection was observed in 18 (14.2%) women and regression in 101 (79.5%). In seven of the women who cleared the HPV16 (7/101; 6.9%) an HPV genotype other than HPV16 was detected. None of these seven women progressed to HSIL/CIN2+.

**Figure 1:**
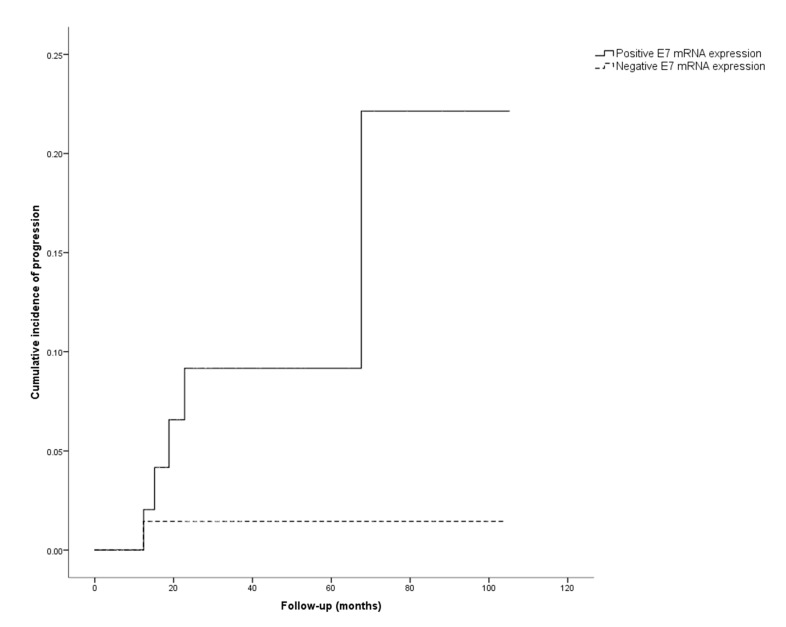
Cumulative incidence of progression to HSIL/CIN2+ in cases with positive HPV16 E7 mRNA expression (solid line) vs. cases with negative HPV16 E7 mRNA (dashed line).

Table 2 shows the clinical characteristics (age, smoking habits), the result of the Pap test histological diagnosis, and the colposcopy findings at the first visit, as well as the results of the HPV16 E7 mRNA expression upon entry in the study in the different outcome groups. Fifty-five out of the 127 (43.3%) women included in the study showed HPV16 E7 mRNA expression. Seven of these women (7/55; 12.7%) developed HSIL/CIN2+ during follow-up. Seventy-two women (56.7%) did not show HPV16 E7 mRNA expression; only one of these women (1.5%) progressed to HSIL/CIN2+. The differences in terms of progression rate between women expressing and not expressing HPV16 E7 mRNA were statistically significant (*p* = 0.027). Figure 1 shows the cumulative incidence of progression to HSIL/CIN2+ according to HPV16 E7 mRNA expression (positive vs. negative). The cumulative risk of progression was higher in women with positive HPV16 E7 mRNA expression (*p* = 0.008).

The risk of progression to HSIL/CIN2+ according to age, Pap smear result, histological diagnosis, colposcopy findings, and HPV16 E7 mRNA expression at the first visit is shown in Table 3. HPV16 E7 mRNA expression was associated with a 10-fold increase in the risk of progression to HSIL/CIN2+ (hazard ratio [HR] 10.0; 95% confidence interval [CI] 1.2–81.4; *p* = 0.031). Age, smoking habit, Pap smear result, histological diagnosis, colposcopy findings, or absence of HPV16 E7 mRNA expression were not significantly associated with the probability of regression (data not shown).

Table 4 shows the clinical and pathological characteristics of the eight women who progressed to HSIL/CIN2+ during follow-up. Biopsy was performed in five (65.5%) cases during follow-up due to a worsening or persistence of the abnormal cytology result and in three cases (37.5%) due to worsening of the colposcopy pattern.

## 4. Discussion

The main finding of this study is that HPV16 E7 mRNA expression is associated with an increased risk of progression in women referred to colposcopy for HPV16 infection in whom a thorough examination with biopsy sampling has excluded HSIL/CIN2+. This biomarker was able to identify 87.5% of the women who progressed. Interestingly, HPV 16 E7 mRNA was negative in almost 60% of these patients and, among these women, only one (1.4%) progressed to HSIL/CIN2+. Thus HPV 16 E7 mRNA would allow less intensive follow-up of women at lower risk of developing a premalignant lesion, selectively assigning the efforts and resources to the 40% of patients who are at higher risk of progression.

Women with HPV16 infection are directly referred to colposcopy due to the higher risk of harboring HSIL/CIN2+. However, there are no clear guidelines on how to proceed with these women once HSIL/CIN2+ has been excluded. In general, they are closely followed during a long time because of the increased risk of developing HSIL/CIN2+, despite only a few developing a premalignant lesion. Indeed, as shown in this study, a high proportion of these women spontaneously cleared HPV infection.

Several factors have been associated with the risk of developing HSIL/CIN2+. These factors include genetic and epigenetic profiles induced by HPV infection [2,6,9,29,30]. E6 and E7 mRNA expression induce abnormal cellular proliferation, transformation, and immortalization, leading to the development of HSIL/CIN2+ by the inhibition of differentiation and immune response [12,13]. Indeed, the usefulness of E7 expression has been extensively evaluated in the diagnosis of HPV-associated lesions. Previous studies have reported that E7 mRNA expression increases with disease grade, suggesting that E7 mRNA may be a good predictor of prevalent HSIL/CIN2+ in HPV-positive women [31,32,33], and the subrogate biomarker of E7 activity, p16, is widely used in the diagnosis of HPV-associated lesions [17]. However, very few studies have determined the value of E7 mRNA expression for identifying women at risk of progression to HSIL/CIN2+. In the present study, we designed a set of primers and probes to specifically allow the identification of HPV16 E7 mRNA expression in women with HPV16 infection. Interestingly, the risk of progression during follow-up of women with positive HPV16 E7 mRNA expression was 10 times higher than that of HPV16 E7 mRNA negative women. The results of our series are in keeping with a previous retrospective study in women with high-risk HPV and minor cytological abnormalities (LSIL or atypical squamous cells of undetermined significance [ASC-US]), which concluded that the presence of HPV E7 mRNA was associated with future development of HSIL/CIN2+ (odds ratio [OR]; 3.9; 95% CI 1.1–20.5) during follow-up [34]. Despite the limitations of this previous study (it did not describe how HSIL/CIN2+ was excluded in the first exam, and data on histological diagnosis during follow-up were retrieved from registers), and the differences with the present series (which included women with high-risk HPV infection different from HPV16), the results reported in terms of prognostic value of E7 mRNA expression were similar to those of the present series. It is remarkable that in the present series, HPV16 E7mRNA was negative in more than half (72/127; 56.7%) of the women, with only one (1.4%) progressing to HSIL/CIN2+. These results are also in keeping with those described in the previously mentioned study [34], suggesting that HPV16 E7 mRNA could be a useful tool to avoid unnecessary intensive follow-up of many women at low risk of progression to HSIL/CIN2+. However, other molecular alterations are likely to be involved in the progression to HSIL/CIN2+, which could explain the progression of the only patient lacking E7 mRNA expression.

In the present series, once HSIL/CIN2+ was adequately ruled out, factors such as age, smoking habits, or the Pap smear result of the first visit did not influence the risk of progression. It is well known that older age, tobacco, or an HSIL result in the Pap smear are factors related to high risk of premalignant lesions and women with these characteristics may benefit from thorough colposcopy and biopsy evaluation (including colposcopy-directed biopsy of abnormal areas, a random biopsy of the transformation zone, in some cases, and endocervical curettage when the transformation zone is not completely visible). However, once a HSIL/CIN2+ is carefully excluded, these women could be managed following the same strategies as those in women with HPV infection and normal cytology or with LSIL/CIN1 [35]. In our study, neither the colposcopy pattern (normal, grade 1 or grade 2 findings) nor the biopsy diagnosis at entry (negative vs. LSIL/CIN1) were associated with the risk of progression. These results are in keeping with previous reports reported by our group [6,36].

The main strength of this study is the prospective design and the long follow-up period (mean 35.2 months). Moreover, a strict follow-up strategy was established, which included liquid-based cytology, HPV testing, and colposcopy every 6 months with directed and/or random biopsies of the transformation zone, and/or endocervical curettage in the case of HSIL result in the Pap test, or significant worsening of the colposcopy pattern, which allowed early diagnosis of progression to HSIL/CIN2+. Another strength of the present series is that our sampling strategy, using liquid-based cytology specimens, allowed different analyses, such as cytology, HPV genotyping, and detection of HPV oncogene transcription to be applied in the same sample to obtain the true status of each woman in each moment of the study.

Our study also has some limitations. The main limitation is the relatively small number of women included, resulting in a small number of patients who progressed to HSIL/CIN2+. This relatively low number of women included is the result of the strict inclusion criteria applied in this study. The main reason for these strict inclusion criteria was to be as accurate as possible in the evaluation of HPV16 E7 mRNA expression, avoiding any risk of cross-reactivity with other HPV genotypes. For that reason, we designed specific primers and probes for HPV16 E7 mRNA and included only women with HPV16 infection in whom HSIL/CIN2+ had been carefully excluded by thorough histologic evaluation (including biopsy of any abnormal pattern as well as random biopsies from normal appearing areas, plus an endocervical curettage when the transformation zone could not be completely assessed) [6]. Another possible limitation is that only one biomarker (HPV16 E7 mRNA expression) was evaluated in the present study. The risk of progression to HSIL/CIN 2+ seems to be a complex, multifactorial process, and consequently, the risk of progression of a particular lesion is probably a combination of several molecular alterations (including E7, E6 mRNA expression, and some specific methylation profiles). Thus, a panel of biomarkers could provide more accurate information on the risk of progression than the evaluation of a single biomarker. Nevertheless, in this study we focused only on E7 mRNA expression, since the true value of a single biomarker should be clearly defined before its use in combination with other markers.

## 5. Conclusions

In conclusion, our study shows that HPV16 E7 mRNA could be a useful biomarker for risk stratification in women with HPV16 infection referred to colposcopy for whom a HSIL/CIN2+ lesion has been ruled out by careful evaluation. The HPV16 E7 mRNA test would allow us to better tailor the management for these women, avoiding an unnecessary intensive follow-up in many women who are at low risk, while identifying the subgroup at higher risk of progression, who would benefit from a closer follow-up. Nevertheless, further studies including a larger number of women and women with HPV infection different from HPV16 are required to confirm the prognostic value of this biomarker in clinical practice.

## Figures and Tables

**Table 1:** Primers and probes used to detect mRNA E7 expression of the HPV types isolated in the cytological samples.

Target Gene	Primers and Probes	Source
E7 HPV	HPV_880_3358,AICSXFL	Life Technologies
E7 HPV	HPV_880_2709,AID1VLT	Life Technologies
E7 HPV	HPV_880_2582,AIFATR1	Life Technologies
GUSB	GUSB (Hs99999908_m1)	Life Technologies
PGK1	PGK1 (Hs99999906_m1)	Life Technologies

**Table 2:** Clinical characteristics and results of biomarkers (HPV genotyping and E7 mRNA) at the first visit of the women included in the study according to their final outcome.

Variables	*n*	Regression (*n* = 101)	Persistence (*n* = 18)	Progression (*n* = 8)	*p*
**Age** (years)	127	36.3 ± 11.2	32.9 ± 10.1	43.3 ± 12.8	0.095
**Smoking habit**					0.921
Non-smoker	59	46 (45.5)	9 (50.0)	4 (50.0)	
Smoker	68	50 (54.5)	9 (50.0)	4(50.0)	
**Cytology at first visit**					0.823
Negative	14	12 (11.9)	2 (11.1)	0 (0.0)	
LSIL	67	54 (53.5)	9 (50.0)	4 (50.0)	
HSIL	46	35 (34.6)	7 (38.9)	4 (50.0)	
**Biopsy at first visit**					0.367
Negative	73	55 (54.5)	12 (66.7)	6 (75.0)	
LSIL/CIN1	54	45 (45.5)	6 (33.3)	2 (25.0)	
**Colposcopy findings at first visit**					0.341
No lesion	27	22 (21.8)	2 (11.1)	3 (37.5)	
Grade 1	82	65 (64.3)	14 (77.8)	3 (37.5)	
Grade 2	18	14 (13.9)	2 (11.1)	2 (25.0)	
**HPV E7 mRNA**					0.027
Negative	72	59 (58.4)	12(66.7)	1 (12.5)	
Positive	55	42 (41.6)	6 (33.3)	7 (87.5)	

Age is presented as mean ± standard deviation. Categorical variables are presented as absolute number and (%); LSIL: low-grade intraepithelial squamous lesion; HSIL: high-grade squamous intraepithelial lesion; CIN: cervical intraepithelial neoplasia; HPV: human papillomavirus; mRNA: messenger RNA.

**Table 3:** Estimation of the risk of progression to high-grade squamous intraepithelial lesion/cervical intraepithelial neoplasia grade 2–3 or cervical carcinoma (HSIL/CIN2+) according to age, smoking habit, colposcopy findings, Pap smear result, histological diagnosis and HPV16 E7 mRNA expression results at first visit.

	Univariate Analysis		
Results at First Visit	HR	(95% CI)	*p*
**Age**			
≤35 years	1		
>35 years	1.8	(0.7–7.4)	0.431
**Smoking habit**			
Non-smoker	1		
Smoker	0.8	(0.3–3.4)	0.803
**Pap smear**			
Negative	1		
LSIL	NA		0.947
HSIL	NA		0.945
**Histological diagnosis**			
Negative	1		
LSIL/CIN1	0.5	(0.1–2.3)	0.340
**Colposcopy findings**			
No abnormal findings	1		
Abnormal findings grade 1	0.3	(0.1–1.6)	0.171
Abnormal findings grade 2	1.1	(0.2–6.7)	0.898
**HPV16 E7 mRNA expression**			
Negative	1		
Positive	10.0	(1.2–81.4)	0.031

HSIL/CIN2+: high-grade squamous intraepithelial lesion/cervical intraepithelial neoplasia grade 2–3 or cervical carcinoma; LSIL: low-grade intraepithelial squamous lesion; HPV16 E7 mRNA: human papillomavirus 16 E7 messenger RNA; HR: Hazard ratio; CI: confidence interval; NA: not applicable (since no women with negative cytology progressed to HSIL/CIN2+, the risk of progression could not be estimated).

**Table 4:** Clinical characteristics of the eight women who progressed to high-grade squamous in Table 2. Or cervical carcinoma (HSIL/CIN2+) during follow-up.

Case	Results at First Visit			Results at Progression		
	Cytology	Biopsy	HPV E7 mRNA	Cytology	Biopsy	Time to Progression *
1	HSIL	Negative	Positive	Negative	HSIL/CIN3	22.8
2	HSIL	Negative	Positive	Negative	HSIL/CIN3	15.1
3	HSIL	CIN1	Positive	HSIL	HSIL/CIN2	14.3
4	HSIL	Negative	Positive	LSIL	HSIL/CIN3	12.8
5	LSIL	Negative	Positive	HSIL	HSIL/CIN2	12.4
6	LSIL	Negative	Negative	HSIL	HSIL/CIN2	12.3
7	LSIL	CIN 1	Positive	HSIL	HSIL/CIN2	18.8
8	LSIL	Negative	Positive	HSIL	HSIL/CIN3	67.6

HPV: Human papillomavirus; mRNA: messenger RNA; LSIL: Low-grade intraepithelial lesion; CIN: Cervical intraepithelial neoplasia. * Time to progression is expressed in months.

## Data Availability

All data collected in this research protocol has been treated confidential. Data protection was performed following EU Regulation 1725/2018. The research databases were compartmentalized (clinical data base and histological/molecular database) and patients’ identification was done by using study codes. The patient identifier wasn’t linked to the specimen codes in the specimen inventory database or research databases. A link between the specimen code(s) and the patient identifier was secured at a trusted third party in a separate secure file only available for the principal investigator. Databases is stored in the hard disk of the center.
